# The Enrichment of Survivin in Exosomes from Breast Cancer Cells Treated with Paclitaxel Promotes Cell Survival and Chemoresistance

**DOI:** 10.3390/cancers8120111

**Published:** 2016-12-09

**Authors:** Bridget T. Kreger, Eric R. Johansen, Richard A. Cerione, Marc A. Antonyak

**Affiliations:** 1 Department of Molecular Medicine, Cornell University, Ithaca, NY 14850, USA; btk37@cornell.edu (B.T.K.); ej95@cornell.edu (E.R.J.); maa27@cornell.edu (M.A.A.); 2 Department of Chemistry and Chemical Biology, Cornell University, Ithaca, NY 14850, USA

**Keywords:** intercellular communication, exosomes, cell signaling, tumor microenvironment, extracellular vesicles, Survivin, chemotherapy, chemoresistance, paclitaxel

## Abstract

The generation and release of membrane-enclosed packets from cancer cells, called extracellular vesicles (EVs), play important roles in propagating transformed phenotypes, including promoting cell survival. EVs mediate their effects by transferring their contents, which include specific proteins and nucleic acids, to target cells. However, how the cargo and function of EVs change in response to different stimuli remains unclear. Here, we discovered that treating highly aggressive MDAMB231 breast cancer cells with paclitaxel (PTX), a chemotherapy that stabilizes microtubules, causes them to generate a specific class of EV, namely exosomes, that are highly enriched with the cell survival protein and cancer marker, Survivin. Treating MDAMB231 cells with a variety of other chemotherapeutic agents, and inhibitors that block cell growth and survival, did not have the same effect as PTX, with the exception of nocodazole, another inhibitor of microtubule dynamics. Exosomes isolated from PTX-treated MDAMB231 cells strongly promoted the survival of serum-starved and PTX-treated fibroblasts and SKBR3 breast cancer cells, an effect that was ablated when Survivin was knocked-down from these vesicles using siRNA. These findings underscore how the enrichment of a specific cargo in exosomes promotes cell survival, as well as can potentially serve as a marker of PTX resistance.

## 1. Introduction

Breast cancer remains one of the most prevalent forms of cancer, with one in eight women being diagnosed with invasive breast cancer in their lifetime [1]. Possible treatment regimens for these patients typically include irradiation, surgery, and/or chemotherapy. However, therapy resistance and tumor recurrence frequently occurs. Thus, there continues to be an overriding need to better understand the mechanisms underlying therapy resistance.

There are several ways that cancer cells have been shown to overcome the cytotoxic effects of chemotherapy [1]. One such mechanism that is starting to attract a good deal of attention involves the ability of cancer cells to generate and release membrane-enclosed packages, collectively referred to as extracellular vesicles (EVs), that contain a wide-range of protein types including cell surface receptors, cytosolic signaling proteins, metabolic enzymes, cytoskeletal components, and nuclear proteins [2,3,4]. EVs also have been shown to contain RNA transcripts, micro-RNAs, and even long non-coding RNAs [5,6,7].

Exosomes and microvesicles (MVs) make-up the two major classes of EVs, and they can be distinguished from one another based on the mechanisms underlying their biogenesis and their size. Exosomes are formed as multivesicular bodies (MVBs) containing intraluminal vesicles which are re-directed from the lysosome, where they would be degraded, to the cell surface [8,9,10]. The MVBs then fuse with the plasma membrane and release their contents, now termed exosomes, into the extracellular space. Exosomes range between 30–100 nm in size. In contrast, MVs are formed through the RhoA- and Arf6-mediated outward budding and fission of the plasma membrane [11,12]. MVs tend to be much larger than exosomes, ranging from 200 nm–2 µm in diameter [3,8,12,13].

Both exosomes and MVs mediate paracrine and endocrine signaling by docking onto cells and transferring their contents into the recipient cells [2]. The addition of EVs derived from highly aggressive cancer cells to other cancer cells, as well as to normal cells, has been shown to enhance their growth and survival [13,14,15]. One of the earliest examples of this came from a study on gliomas. Many of these high grade and aggressive brain tumors were shown to release, or shed, EVs that contained a highly oncogenic form of the epidermal growth factor receptor (EGFR), called EGFR variant type III (EGFRvIII) [3]. The EV-mediated transfer of this mutant receptor to other glioma cells that lacked EGFRvIII expression promoted the activation of Erk1/2 and Akt signaling pathways and increased their rates of growth and survival.

Paclitaxel (PTX), also referred to as taxol, is a frontline treatment for aggressive and high grade forms of breast, lung, bladder, prostate, and ovarian cancer [16]. It works by interfering with the ability of microtubules to undergo normal cycles of polymerization and disassembly [17]. Specifically, PTX binds to microtubules and stabilizes them, an outcome that causes dividing cells to undergo cell cycle arrest and die [18,19].

Intrinsic or acquired resistance to PTX, like most cancer therapies, is a major hurdle confronted by oncologists [1,20]. Here, we investigated whether EVs could potentially contribute to PTX resistance. We discovered that treating the aggressive MDAMB231 breast cancer cell line with PTX causes them to generate exosomes that are highly enriched with Survivin, a protein whose expression is tightly correlated with poor patient prognosis, chemotherapy resistance, and tumor recurrence [21,22,23,24]. Interestingly, Survivin was not detected in the larger class of EVs generated by these cancer cells (i.e., the MVs), nor was it enriched to the same extent in exosomes collected from MDAMB231 treated with a variety of other chemotherapeutic agents and inhibitors known to block cell growth and survival. We then went on to show that the ability of exosomes from PTX-treated MDAMB231 cells to strongly promote the survival of fibroblasts and SKBR3 breast cancer cells challenged with serum-starvation or PTX treatment could be eliminated by knocking-down Survivin expression from these vesicles using siRNA. Thus, these findings suggest that at least a portion of the resistance to PTX encountered in the clinics could be due to the generation of exosomes that are uniquely enriched with a specific cargo.

## 2. Results

### 2.1. Cancer Cells Shed Exosomes that Promote Cell Survival

**Figure 1:**
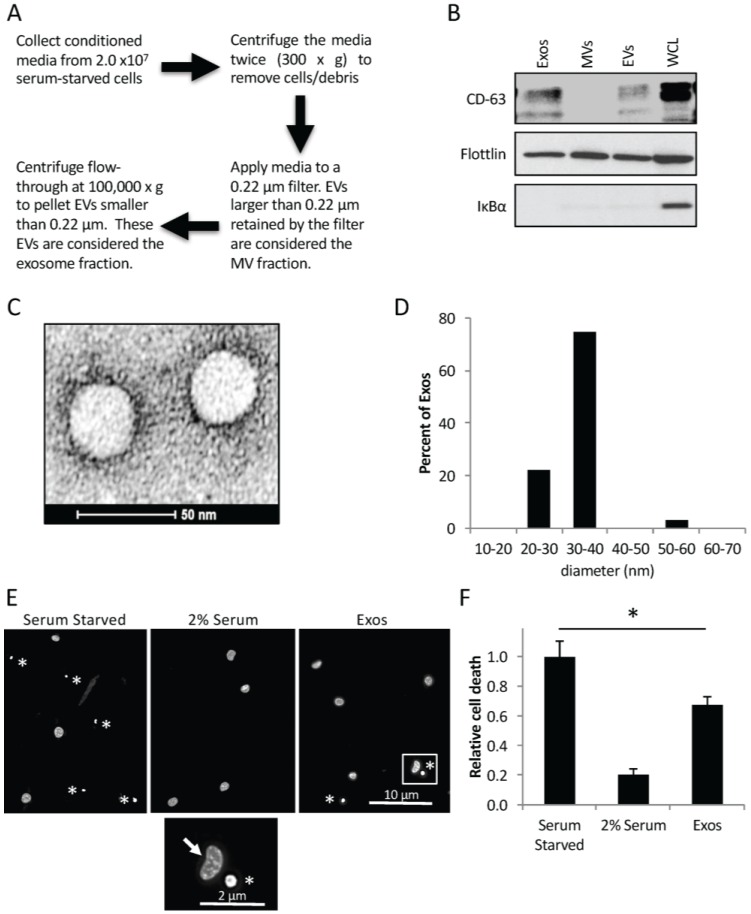
MDAMB231 breast cancer cells shed exosomes and exosomes and microvesicles (MVs). (**A**) Outline of procedure used to isolate exosomes and MVs from conditioned medium. (**B**) Western blot analysis using CD-63, IκBα, and flotillin antibodies was performed on lysates of MDAMB231 cells (lane labeled WCL), and the exosomes (lane labeled Exos), and MVs (lane labeled MVs) that these cells generated. A sample containing all extracellular vesicles (EVs) (including both MVs and exosomes) generated by the cells (lane labeled EVs) was also included on the blot. (**C**) Transmission electron microscopy (TEM) image of exosomes isolated from MDAMB231 cells. Scale bar = 50 nm. (**D**) Histogram showing the sizes of exosomes detected in C. (**E**,**F**) NIH-3T3 fibroblasts were cultured in serum-free media supplemented without (images and bars labeled Serum Starved) or with either 2% serum (images and bars labeled 2% Serum), or 0.5 × 10^6^ exosomes/mL collected from MDAMB231 cells (images and bars labeled Exos) for two days, at which point the cells were stained with DAPI to label nuclei. (**E**) Representative fluorescent images of the nuclei from cells cultured under each of the indicated conditions. Asterisks indicate condensed/blebbed nuclei, a hallmark of apoptosis. Scale bar = 10 μm. The boxed portion of the fibroblasts treated with exosomes (Exos) represents an enlarged image and was placed below the other images to further highlight the differences between a normal (non-apoptotic) nuclei (indicated with an arrow) and an apoptotic nuclei (indicated with an asterisk). Scale bar = 2 μm (**F**) The results of this cell death assay were quantified. At least 300 nuclei were counted for each condition analyzed. The experiments in D and F were performed a minimum of three separate times, with each repeat yielding similar results. The data shown represents the mean ± standard deviation (SD). Student *t*-tests were performed. * *p* < 0.05.

We began by determining the amount and size of exosomes that are inherently generated by the triple negative MDAMB231 breast cancer cell line. The conditioned medium from 2.0 × 10^7^ serum-starved MDAMB231 cells was collected, and the exosomes and MVs were isolated using an approach that involves a series of centrifugation and filtration steps (Figure 1A). The individual exosome and MV preparations, a sample containing both types of EVs, as well as the MDAMB231 cells, themselves, were lysed and then Western blotted using an antibody that recognizes the exosomal marker CD-63. Figure 1B (top panel) shows that CD-63 could be detected in both the exosomes (lane labeled Exos), and in the sample containing both types of EVs (lane labeled EVs), but not in the MV preparation (lane labeled MVs). The blot was also probed for flotillin and IκBα. Flotillin is a general EV marker, and was detected in each sample (middle panel). In contrast, the cytosolic signaling protein IκBα was only seen in the cell lysates (bottom panel, lane labeled WCL), suggesting that we not only reliably separated exosomes from MVs, but our different EV preparations lacked cytosolic contaminants.

Additional batches of exosomes from MDAMB231 cells were collected and analyzed by transmission electron microscopy (TEM). Many vesicular structures were detected (Figure 1C), with a large majority of them (~75%) averaging between 30–40 nm in diameter (Figure 1D), consistent with the known size of exosomes [9,10].

The exosomes generated by MDAMB231 cells were then assayed for their ability to promote cell survival. Culturing cells in medium lacking serum is a stress known to induce cell death [11,13]. Indeed, we found that ~60% of NIH-3T3 fibroblasts that had been serum-starved died, as read-out by the appearance of condensed and/or blebbed nuclei (Figure 1E,F), a distinct trait of apoptotic cells [11,13]. This outcome could be blocked by the addition of a small amount of serum (2% serum) to the medium (Figure 1F, compare bars 1 and 2). Fibroblasts cultured in serum-free medium supplemented with 0.5 × 10^6^ exosomes/mL isolated from MDAMB231 cells, showed a ~30% reduction in cell death (Figure 1F, compare bars 1 and 3).

### 2.2. Exosomes from PTX-Treated MDAMB231 Cells Strongly Promote Cell Survival

**Figure 2:**
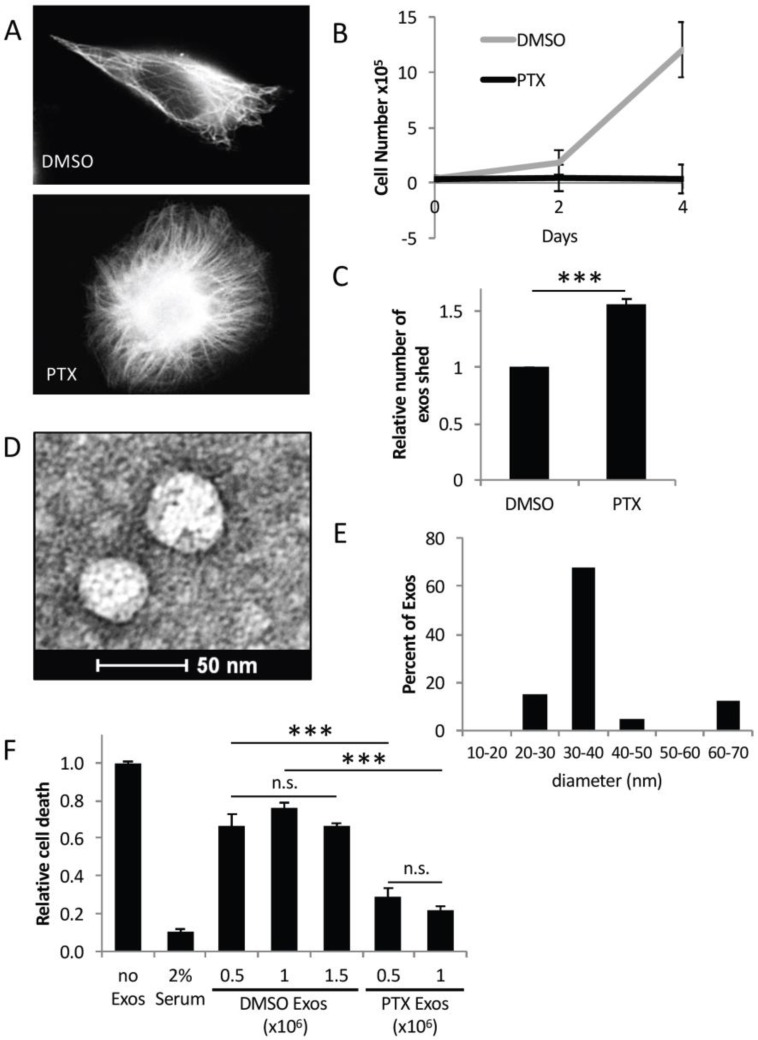
Exosomes from PTX-treated MDAMB231 cells strongly promote cell survival. (**A**) Immunofluorescence using a tubulin antibody was performed on MDAMB231 cells treated with either DMSO (top image), or 50 nM PTX (bottom image), for 8 h. Scale bar = 10 µm. (**B**) Cell growth assays were performed on MDAMB231 cells treated with either DMSO (grey line) or 50 nM PTX (black line). (**C**) The relative amounts of exosomes generated by DMSO- or PTX-treated MDAMB231 cells were determined using nanoparticle tracking analysis (NTA). (**D**) TEM image of exosomes isolated from MDAMB231 cells treated with PTX. Scale bar = 50 nm. (**E**) Histogram showing the sizes of exosomes detected in (**D**). (**F**) Cell death assays were performed on NIH-3T3 fibroblasts cultured in serum-free media supplemented without (bar labeled Serum Starved) or with 2% serum (bar labeled 2% Serum), or with the indicated amounts of exosomes from DMSO-treated (bars labeled DMSO Exos) or PTX-treated (bars labeled PTX Exos) MDAMB231 cells. The experiments in (**B,C,E,F**)were performed a minimum of three separate times, with each experiment yielding similar results. The data shown represents the mean ± SD. Student *t*-tests were performed. *** *p* < 0.001; n.s., not significant.

Since PTX is used to treat patients with breast cancer [1,16,20], we wanted to see whether this drug influenced the biogenesis and function of exosomes generated by MDAMB231 breast cancer cells. Thus, multiple sets of this cell line were treated without dimethyl sulfoxide (DMSO) or with 50 nM PTX, an amount of the chemotherapeutic drug routinely used to treat cancer cells [18,19]. Immunofluorescence microscopy using a tubulin antibody to detect microtubules was performed on one set of the cells. Figure 2A shows that MDAMB231 cells treated with DMSO exhibited a typical polarized morphology with microtubules traversing the cell (top panel). However, PTX treatment caused the cells to lose their polarity (bottom panel). This morphological change was accompanied by a large increase in the amount of microtubules present in the MDAMB231 cells, consistent with PTX being a microtubule stabilizing drug [16,25]. A cell growth assay performed on another set of MDAMB231 cells treated with either DMSO or 50 nM PTX showed that the growth of the cancer cells was completely ablated by the drug treatment (Figure 2B). Thus, 50 nM PTX was used to treat the various cancer cell lines throughout the study.

Next, we determined how exosome biogenesis by MDAMB231 cells was impacted by PTX treatment. An equivalent number of cells were treated with either DMSO or PTX, and the exosomes that they generated were isolated and subjected to nanoparticle tracking analysis (NTA) to determine the amount of exosomes present in each sample. Interestingly, PTX-treated MDAMB231 cells consistently generated ~1.5-fold more exosomes compared to cells treated with DMSO alone (Figure 2C). TEM performed on these same exosome preparations revealed that most of the exosomes derived from PTX-treated MDAMB231 cells were similar in size (i.e., ~30–40 nm) to those generated by control MDAMB231 cells (compare Figure 2D,E to Figure 1C,D).

The ability of exosomes from MDAMB231 cells treated with PTX to promote cell survival was then assayed. The exosomes collected from DMSO- or PTX-treated cells were normalized based on the NTA results (see Figure 2C), and added at increasing amounts to cultures of serum-starved NIH-3T3 fibroblasts (Figure 2F). The fibroblasts were re-treated the next day with freshly prepared exosomes, and then two days after the start of the assay, the cells were analyzed for cell death, as read-out by the appearance of condensed/blebbed nuclei. Figure 2F shows that serum-starved fibroblasts underwent a high level of death, compared to cells maintained in medium supplemented with 2% serum (compare bars 1 and 2) (i.e., the positive control). Fibroblasts incubated with 0.5 × 10^6^ exosomes/mL isolated from MDAMB231 cells treated with DMSO, again exhibited reduced levels of cell death caused by serum starvation by ~30% (compare bars 1 and 3). Increasing the concentration of these exosomes from 0.5 × 10^6^ exosomes/mL to 1.5 × 10^6^ exosomes/mL did not further reduce the amount of cells that died (compare bars 3–5), suggesting that a maximal effect was attained with 0.5 × 10^6^ exosomes/mL. However, when the same experiment was performed with exosomes derived from MDAMB231 cells treated with PTX, a much stronger survival advantage was observed. Indeed, between a 70%–80% reduction in serum starvation-induced NIH3T3 cell death was achieved by the addition of 0.5–1.0 × 10^6^ exosomes/mL isolated from PTX-treated cancer cells to the culturing media. (Figure 2F, compare bars 1, 6, and 7).

### 2.3. PTX-Treated Cells Generate Exosomes Enriched with Survivin

**Figure 3:**
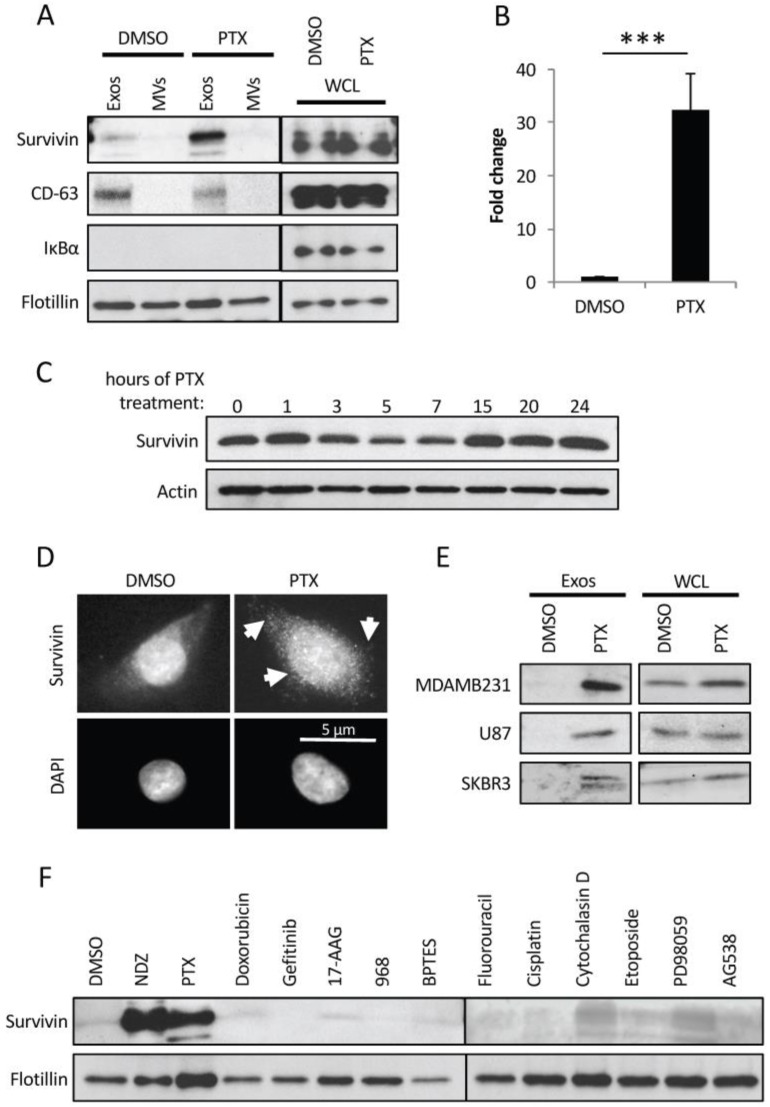
Survivin is highly enriched in exosomes from PTX-treated cancer cells. (**A**) Western blot analysis using Survivin, flotillin, IκBα, and CD-63 antibodies was performed on lysates of MDAMB231 cells treated with either DMSO or PTX (lanes labeled WCL), as well as the exosomes (lanes labeled Exos) and MVs (lanes labeled MVs) generated by the cells. (**B**) The relative amounts of Survivin detected in exosomes generated by DMSO- and PTX-treated MDAMB231 cells. (**C**) Western blot analysis using Survivin and actin antibodies was performed on lysates of MDAMB231 cells treated with PTX for increasing lengths of time. (**D**) Immunofluorescence using a Survivin antibody was performed on MDAMB231 cells treated with either DMSO or PTX (top images). The cells were also stained with DAPI to label nuclei (bottom images). Arrows indicate areas where Survivin is detected as puncta in the cytosol of cells treated with PTX. Scale bar = 5 µm. (**E**) Western blot analysis was performed using a Survivin antibody on lysates of MDAMB231 cells, U87 glioblastoma cells, and SKBR3 cells that had been treated with DMSO or PTX (lanes labeled WCL), and on the exosomes these cells generated (lanes labeled Exos). (**F**) Western blot analysis was performed on lysates of exosomes from MDAMB231 cells that had been treated with the indicated chemotherapeutic agents and inhibitors. The experiments in *B* were performed a minimum of three separate times, with each experiment yielding similar results. Student t-tests were performed. *** *p* < 0.001.

These findings suggested that there is something unique about the contents of exosomes from PTX-treated MDAMB231 cancer cells, which enables them to promote enhanced survival. To identify the cargo responsible for conferring this beneficial effect, lysates of exosomes and MVs prepared from DMSO- or PTX-treated MDAMB231 cells were Western blotted for several different proteins known to be involved in promoting cell survival. One protein whose expression consistently changed in exosomes from PTX-treated cells was Survivin. Figure 3A shows that Survivin is present at low levels in exosomes from DMSO-treated control cells (top panel, lane 1). However, its expression increased sharply in exosomes from MDAMB231 cells treated with PTX (top panel, lane 3). Specifically, we observed a dramatic ~30-fold increase in the amount of Survivin detected in exosomes prepared from PTX-treated cells compared to exosomes isolated from control cells (Figure 3B). Moreover, we found that the expression of Survivin was specific for exosomes, as the larger MVs isolated from the same PTX-treated MDAMB231 cells lacked detectable levels of this protein (Figure 3A, top panel, lane 4). The cellular levels of Survivin did not change in response to PTX treatment in this experiment (Figure 3A, compare lanes 5 and 6), while its expression decreased slightly before recovering to the levels observed in untreated control cells in experiments where MDAMB231 cells were incubated with the chemotherapeutic drug for increasing lengths of time (Figure 3C, top panel). However, we did find that the localization of Survivin consistently changed in MDAMB231 cells treated with PTX. Specifically, while Survivin was predominantly localized in the nucleus of control (DMSO-treated) MDAMB231 cells (Figure 3D, upper left panel), its localization changed in PTX-treated cells, such that it appeared as small puncta throughout the cytosol (Figure 3D, upper right panel, see arrows). These findings suggest that the enrichment of Survivin in exosomes generated by PTX-treated MDAMB231 cells cannot be directly attributed to changes in the expression levels of this protein in cells, but rather may be due to its redistribution to the cytosol.

We then examined how the levels of Survivin in exosomes generated by two other cancer cell lines change in response to PTX. The U87 glioblastoma cell line and the SKBR3 breast cancer cell line, along with MDAMB231 cells, as a control, were treated without (DMSO alone) or with PTX. The exosomes that each of these cell cultures generated, as well as the cells themselves, were collected and analyzed for Survivin expression. Figure 3E shows that while Survivin is expressed in each of these cancer cell lines (compare right panels), it was only detected in exosomes produced by U87 and SKBR3 cells following their treatment with PTX, similar to MDAMB231 cells (compare left panels).

We asked whether other chemotherapeutic agents, as well as inhibitors known to interfere with the function of proteins that are important for maintaining the transformed phenotype, cause a similar enrichment of Survivin in exosomes as that induced by PTX. Thus, MDAMB231 breast cancer cells were treated with another microtubule disruptor (nocodazole), a MEK inhibitor (PD98059), the metabolic inhibitors BPTES and 968 (which allosterically block the activation of the mitochondrial enzyme glutaminase), various DNA synthesis inhibitors (doxorubicin, fluorouracil, etoposide, and cisplatin), receptor tyrosine kinase inhibitors (Gefitinib and AG538), an inhibitor of actin polymerization (cytochalasin D), and a heat shock protein (HSP)90 inhibitor (17-AAG). DMSO- and PTX-treatments were used as controls. Lysates of the exosomes generated from cells treated with each of the drugs/inhibitors were first Western blotted using a flotillin antibody. Figure 3F shows that roughly equivalent amounts of this EV marker was detected in each of the samples, suggesting that the generation of exosomes by the cancer cells was not blocked by any of the treatments (bottom panel). However, when the same lysates were probed for Survivin, striking differences were observed. Although many of the chemotherapeutic agents and inhibitors caused small increases in the levels of Survivin in exosomes, none of them matched the effects of PTX, which caused a dramatic enrichment in the levels of Survivin in the vesicles, with the exception of the microtubule disruptor nocodazole (Figure 3F, top panel). This suggests that the enrichment of Survivin in exosomes derived from MDAMB231 breast cancer cells is not a general outcome of stressing cancer cells. Rather, it appears to occur specifically when normal microtubule dynamics are disrupted.

### 2.4. Survivin in Exosomes Promotes Cell Survival

**Figure 4:**
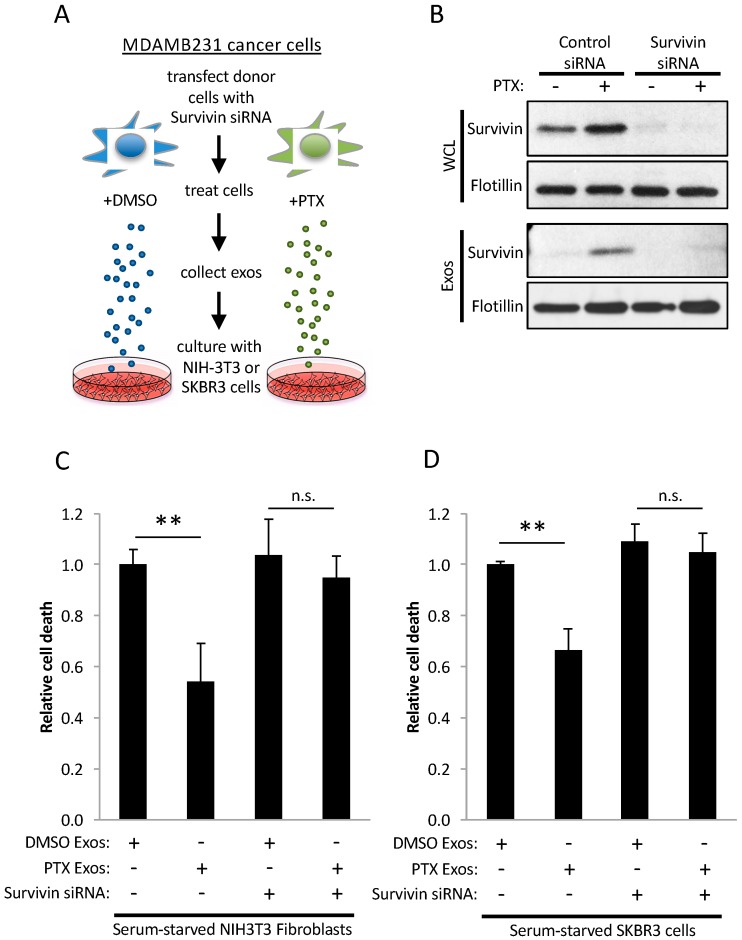
Survivin is important for the strong cell survival-promoting activities of exosomes from MDAMB231 cells treated with PTX. (**A**) Schematic of the cell death assays performed. Serum-starved NIH-3T3 fibroblasts, or SKBR3 breast cancer cells, were treated with exosomes derived from DMSO-treated (DMSO Exos) or PTX-treated (PTX Exos) MDAMB231 cells ectopically expressing either control siRNA (bar labeled—under Survivin siRNA), or a Survivin-specific siRNA (Survivin siRNA), as indicated. The relative amount of cell death that occurred for each culturing condition was determined. (**B**) Western blot analysis using Survivin and flotillin antibodies was performed on lysates of DMSO- and PTX-treated MDAMB231 cells ectopically expressing either control siRNA or Survivin siRNA (panels labeled WCL), as well as on the exosomes these cells generated (panels labeled Exos). (**C**,**D**) Results of the cell death assay described in (**A**) using (**C**) NIH-3T3 fibroblasts, and (**D**) SKBR3 breast cancer cells, as the recipient cells. The experiments were performed a minimum of three separate times, with each experiment yielding similar results. The data shown represents the mean ± SD. Student *t*-tests were performed. ** *p* < 0.01; n.s., not significant.

An experiment was performed (Figure 4A) to determine whether the enrichment of Survivin in exosomes from PTX-treated MDAMB231 cells was responsible for their strong cell survival-promoting capabilities. MDAMB231 cells expressing control siRNA, or a Survivin-specific siRNA, were treated with either DMSO or PTX. The Survivin-specific siRNA reduced the levels of Survivin expression in the cells by greater than 90% (Figure 4B, panels labeled WCL) and, correspondingly, in the exosomes derived from the PTX-treated cancer cells (Figure 4B, panels labeled Exos). The ability of the exosomes from the PTX-treated cells, depleted of Survivin, to promote cell survival was then assessed. Figure 4C shows again that exosomes from PTX-treated cells are far better at preventing the death of serum-starved NIH-3T3 fibroblasts compared to exosomes from DMSO-treated control cells (compare bars 1 and 2). However, this benefit was lost when Survivin expression was depleted from these exosomes by siRNA (compare bars 2 and 4). Similar results were obtained when the same cell survival experiment was performed using the less aggressive SKBR3 breast cancer cell line as the recipient cells (Figure 4D).

### 2.5. Exosomes Derived from PTX-Treated Cells Promote Chemoresistance

**Figure 5:**
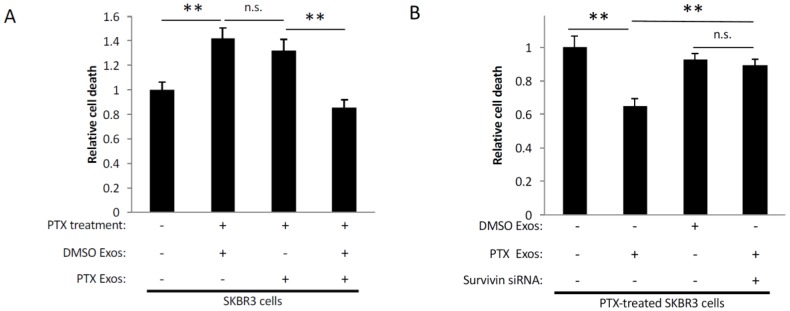
Exosomes derived from PTX treated cancer cells promote chemoresistance. (**A**) Serum-starved SKBR3 breast cancer cells treated with either DMSO (bar labeled—under PTX treatment) or PTX (PTX treatment), were further treated with the same exosome preparations derived from DMSO-treated (DMSO Exos) or PTX-treated (PTX Exos) MDAMB231 cells shown in Figure 4B. The amount of cell death that occurred for each culturing condition was determined. (**B**) Serum-starved SKBR3 cells treated with PTX were further treated with exosomes derived from DMSO-treated (DMSO Exos) or PTX-treated (PTX Exos) MDAMB231 cells ectopically expressing control siRNA (bars labeled—under Survivin siRNA) or a Survivin-specific siRNA (Survivin siRNA), as indicated. The relative amount of cell death that occurred for each culturing condition was determined. The experiments were performed a minimum of three separate times, with each experiment yielding similar results. The data shown represents the mean ± SD. Student *t*-tests were performed. ** *p* < 0.01; n.s., not significant.

We expanded upon these findings by determining whether exosomes from MDAMB231 cells treated with PTX could also promote chemoresistance, as might occur within a tumor when a patient is administered this chemotherapeutic agent. We first demonstrated that incubating SKBR3 breast cancer cells with 50 nM PTX caused them to undergo apoptosis (Figure 5A, compare bars 1 and 2). Then, using the same exosome preparations isolated in Figure 4B, we showed that incubating SKBR3 cells with exosomes from control (DMSO-treated) MDAMB231 cells did not change their sensitivity to PTX (compare bars 2 and 3). However, when SKBR3 cells were incubated with both PTX and exosomes collected from MDAMB231 cells that had been treated with PTX, the resulting amount of cell death was reduced to the levels observed in SKBR3 cells that were not treated with the drug (compare bars 1 and 4). The importance of Survivin in mediating this effect was then demonstrated by adding exosomes from PTX-treated MDAMB231 cells expressing Survivin siRNA to SKBR3 cells challenged with PTX. Figure 5B shows that the loss of Survivin from these exosomes eliminated their protective effect.

## 3. Discussion

EVs have been garnering a good deal of attention over the past several years due to their ability to mediate cell-to-cell communication events that influence a wide-range of cellular outcomes, especially as they relate to cancer progression [2,3,4,5]. Both of the major classes of EVs, exosomes and MVs, have been strongly associated with helping maintain the transformed phenotype. For example, colon cancer cells expressing an activated mutant form of KRAS have been shown to generate exosomes that are capable of enhancing the anchorage-independent growth and invasive activity of other colon cancer cell lines [26]. Moreover, we recently found that MVs generated by mouse embryonic fibroblasts induced to express an oncogenic form of the diffuse B-cell lymphoma (Dbl) protein, a potent activator of members of the Rho family of small GTPases [27], dramatically changed the behavior of recipient cells. In particular, we showed that when these MVs were isolated, and then added to cultures of naïve fibroblasts, they induced a transformed-like phenotype in the cells that included promoting their survival and inducing their ability to grow under anchorage-independent (soft agar) conditions [28].

Here, we considered the role played by EVs in promoting cell survival and chemoresistance. Specifically, we were interested in seeing whether PTX, a chemotherapeutic agent used as a frontline treatment for a variety of cancer types [1,16,20], influenced the biogenesis and function of exosomes generated by breast cancer cells. We discovered that treating the highly aggressive MDAMB231 breast cancer cell line with PTX not only increased the amount of exosomes that these cells generated, but it also caused an enrichment in the amount of Survivin present as cargo in the vesicles, an effect that had important functional consequences. Namely, these exosomes strongly promoted the survival of fibroblasts, as well as SKBR3 breast cancer cells, challenged with serum starvation or PTX treatment. These findings suggest that when a breast cancer patient is being administered PTX as a therapy, the cancer cells within a tumor will begin to generate increased amounts of exosomes, as well as increase the levels of Survivin in these vesicles. These exosomes can then be transferred to other cancer cells, or to normal cells, that comprise the tumor microenvironment, and promote their survival. Thus, exosomes might play an important role in mediating resistance to PTX. These findings also raise the exciting possibility that combining PTX with inhibitors of exosome biogenesis might offer a potential combination therapy that could overcome PTX resistance and increase the efficacy of this chemotherapeutic drug. 

Although we do not fully understand the mechanism underlying the enrichment of Survivin in exosomes from cancer cells treated with PTX, we do know that this outcome appears to be rather specific. For example, Survivin is not present in the larger class of EVs, referred to as MVs. To date, differences in the functional consequences of MVs versus exosomes have not been well established. However, the fact that a certain protein known to promote cancer progression (i.e., Survivin) is preferentially expressed in only one class of EV raises the interesting possibility that MVs and exosomes might mediate distinct biological outcomes.

There is also specificity regarding the stimuli that cause the recruitment of Survivin to exosomes. MDAMB231 cells treated with a wide-range of chemotherapeutic agents, as well as various inhibitors known to interfere with the function of proteins that promote cell growth and survival, failed to produce exosomes that contained high levels of Survivin, like PTX, with the exception of nocodazole. This is potentially interesting, as both PTX and nocodazole work by disrupting the normal functioning of microtubules, but they do so in different ways. PTX stabilizes microtubule formation [1,16,20], while nocodazole inhibits their assembly [29]. Based on these findings, we are beginning to favor the idea that drugs which disrupt normal microtubule dynamics (i.e., PTX) cause them to generate exosomes uniquely enriched with Survivin. We are now well-positioned to further determine the mechanisms that underlie this interesting effect.

## 4. Materials and Methods

### 4.1. Cell Culture and Transfections

MBAMB231, SKBR3, and U87 cells were maintained in RPMI (Roswell Park Memorial Institute) medium containing 10% fetal bovine serum, while NIH-3T3 cells were grown in DMEM (Dulbecco’s Modified Eagle’s Medium) containing 10% calf serum. Signal Silence Control siRNA (Cell Signaling, Catalog No. 6568) and Signal Silence Survivin siRNA II (Cell Signaling, Catalog No. 6546) were introduced into cells with Lipofectamine 2000 (Invitrogen, Carlsbad, CA, USA). Cells were treated with 50 nM PTX (Sigma, St. Louis, MO, USA), 1 µM nocodazole (Sigma), 5 µM PD98059 (Cell Signaling, Danvers, MA, USA), 10 µM BPTES (EMD Millipore, Darmstadt, Germany), 10 µM 968 (EMD Millipore), 2 µM doxorubicin (Sigma), 5 µM fluorouracil (Sigma), 5 µM etoposide (Sigma), 2 µM cisplatin (Sigma), 2 µM Gefitinib (LC Laboratories, Woburn, MA, USA), 5 µM AG538 (EMD Millipore), 1 µM cytochalasin D (Sigma), and 5 µM 17-AAG (Sigma).

### 4.2. Immunofluorescence

Cells grown on glass coverslips were treated with either DMSO or PTX for 10 h and fixed with 3.7% formaldehyde. The cells were permeabilized with phosphate buffered saline (PBS) containing 0.1% Triton-X100, and blocked with 10% bovine serum albumin diluted in PBS. The cells were incubated with a Survivin (Catalog No. NB500-201; Novus Biologicals, Littleton, CO, USA) or tubulin antibody (Cell Signaling, Catalog No. 2148). The primary antibodies were detected using an Alexa Fluor 488-conjugated secondary antibody (Catalog No. A-11034; Molecular Probes, Eugene, OR, USA), and the cells were also stained with DAPI (Sigma) to label nuclei. Digital images of the cells were captured using a Zeiss fluorescence microscope outfitted with a Sensicam qe camera (Cooke Co., Campbell, CA, USA) and processed using IPLABS software (BD Biosciences, San Jose, CA, USA).

### 4.3. Isolation of Exosomes and MVs

The conditioned media collected from 2.0 × 10^7^ serum-starved cells, that had been treated as indicated, was subjected to two consecutive centrifugations at 300× *g* to clarify the media of intact cells and debris. The partially clarified media was then filtered using a Steriflip PVDF (polyvinylidene fluoride) filter with a 0.22 µm pore size (Millipore). The EVs retained by the filter (i.e., those larger than 0.22 µm in diameter) were rinsed extensively with PBS before being lysed with lysis buffer (25 mM Tris, 100 mM NaCl, 1% Triton X-100, 1 mM EDTA, 1 mM DTT, 1 mM NaVO_4_, 1 mM β-glycerol phosphate, and 1 µg/mL each of aprotinin and leupeptin). This is considered the MV lysate. The medium and PBS washes that flowed through the filter were centrifuged at 100,000× *g* for two hours to pellet the exosomes. These pellets were either resuspended in serum-free medium for the cell-based assays, TEM, and NTA, or lysed using lysis buffer. Whole cell lysates (WCLs) were prepared by rinsing dishes of cells with PBS, adding lysis buffer, and scraping the cells off of the dish. The resulting lysates were centrifuged at 17,500× *g* for 10 min, and then the supernatants were analyzed.

### 4.4. Immunoblot Analysis

The protein concentrations of cell and EV lysates were determined using the Bio-Rad DC protein assay (Bio-Rad, Hercules, CA, USA). The lysates were normalized by protein concentration, resolved by SDS-PAGE, and then the proteins were transferred to PVDF membranes. The membranes were incubated with various primary antibodies including β-actin (Catalog No. ab8226; Abcam, Cambridge, MA, USA), Survivin (Catalog No. NB500-201; Novus Biologicals), flotillin-2 (Catalog No. 3436S; Cell Signaling), CD-63 (Catalog No. 10628D; ThermoFisher, Waltham, MA, USA), and IκBα (Catalog No. 9242; Cell Signaling), diluted in in 20 mM Tris, 135 mM NaCl, and 0.02% Tween 20 (TBST). The primary antibodies were detected with HRP-conjugated secondary antibodies (Catalog Nos. 7074S and 7076S; Cell Signaling) followed by exposure to ECL (enhanced chemiluminescence) reagent (Catalog No. 32106; ThermoFisher).

### 4.5. Cell Death Assay

NIH-3T3 fibroblasts and SKBR3 breast cancer cells were plated in each well of a six-well dish and cultured in serum-free medium without (serum-starved), or with various combinations of 2% serum, 0.5 × 10^6^–1.5 × 10^6^ exosomes/mL from DMSO- or PTX-treated MDAMB231 breast cancer cells, DMSO, and PTX. For all of the conditions involving exosomes, the cells were re-treated with another dose of freshly prepared exosomes the following day. One day later for the NIH-3T3 fibroblasts, and four days later for the SKBR3 cells, the cells were collected, stained with DAPI, and viewed using fluorescent microscopy. Cells undergoing apoptosis were identified by nuclear condensation or blebbing and the percentage of cell death was calculated by determining the ratio of apoptotic cells to total cells for each condition. At least 300 nuclei were counted for each condition analyzed.

### 4.6. Cell Growth Assay

MDAMB231 cells were plated in each well of a six-well dish at a density of 10 × 10^4^ cells/well and maintained in RPMI medium containing 1% serum, supplemented without (DMSO alone) or with 50 nM PTX. Every other day for four days, one set of cultures was collected and counted.

### 4.7. NTA

The amount of exosomes in a sample was determined using a NanoSight NS300 (Malvern, Malvern, UK). The samples were diluted in PBS made from ultra-pure water and passed through the beam path and detected as points of diffracted light moving rapidly under Brownian motion. Five 60 s digital videos of the exosomes in a sample were captured and analyzed to determine exosome concentrations.

### 4.8. TEM

Isolated exosomes were added to a carbon-coated, 300-mesh copper grid and then stained with 1.75% uranyl acetate. Once dry, the samples were imaged using the FEI T12 Spirit 120 kV field emission TEM at Cornell’s Center for Materials Research (CCMR), supported by NSF MRSEC award number: NSF DMR-1120296.

### 4.9. Statistical Analysis

All experiments were performed a minimum of three independent times, with each experiment yielding similar results. Many of the results shown were presented as histograms or plots with mean and standard deviation (SD). Student *t*-tests were perfomed to assess statistical significance in all cases.

### 4.10. Ethical Statement

The MBAMB231, SKBR3, U87, and NIH-3T3 cell lines were purchased from the American Type Culture Collection (ATCC).

## 5. Conclusions

Intrinsic or acquired resistance to the chemotherapeutic agent PTX, like most cancer therapies, is a major hurdle confronted by oncologists. We discovered that treating cancer cells with PTX causes them to generate a specific class of EVs, namely exosomes, that are uniquely enriched with Survivin, a protein whose expression is tightly correlated with poor patient prognosis, chemotherapy resistance, and tumor recurrence. These exosomes are capable of strongly promoting the survival of fibroblasts and other cancer cells challenged with serum-starvation or PTX treatment, an effect that was ablated by knocking-down Survivin expression from these vesicles using siRNA. Thus, these findings highlight a potentially novel mechanism of PTX-resistance, which involves the generation of exosomes that are uniquely enriched with a specific cargo.
